# Red Cell Distribution Width–Standard Deviation and the Severity of In-Stent Restenosis: Associations with Angiographic Stenosis Burden and Mehran Classification

**DOI:** 10.3390/medicina62071358

**Published:** 2026-07-14

**Authors:** Mert Deniz Savcilioglu, Kemal Ozan Lule, Osman Buyukcelebi, Ertan Vuruskan

**Affiliations:** 1 Cardiology Department, Faculty of Medicine, Gaziantep University, Şahinbey 27410, Türkiye; dr.buyukcelebi02@gmail.com (O.B.); ertanvuruskan@hotmail.com (E.V.); 2 Internal Medicine Department, Faculty of Medicine, Gaziantep University, Şahinbey 27410, Türkiye; drkemalozanlule@gmail.com

**Keywords:** in-stent restenosis, RDW-SD, red cell distribution width, Mehran classification, drug-eluting stent, restenosis severity

## Abstract

*Background and Objectives*: Red cell distribution width–standard deviation (RDW-SD) has been associated with systemic inflammation and adverse cardiovascular outcomes, but its relationship with the angiographic severity and morphological complexity of drug-eluting stent-in-stent restenosis (ISR) has not been systematically characterized. The present study investigated whether RDW-SD is associated with angiographic restenosis severity and restenotic lesion complexity, and compared its performance with the platelet distribution width (PDW), Metabolic Stress Index (MSI), and Platelet-to-HDL Ratio (PHR). *Materials and Methods*: In this retrospective single-center observational study, 290 patients undergoing clinically indicated repeat coronary angiography following prior drug-eluting stent (DES) implantation were enrolled. Angiographic luminal narrowing was quantified by QCA and categorized as reference (<50% in-stent luminal narrowing; *n* = 111), intermediate ISR (50–69%; *n* = 76), and severe ISR (≥70%; *n* = 103). The Mehran classification was applied to patients with ISR ≥50% and dichotomized as Mehran class I–II (*n* = 91) vs. Mehran class III–IV (*n* = 70). Multivariable logistic regression, hierarchical modeling, and incremental discrimination analyses (IDI and NRI) were performed for both binary outcomes. *Results*: RDW-SD differed significantly across angiographic severity groups (Kruskal–Wallis H = 51.14, *p* < 0.001), being highest in the ISR ≥70% group [44.6 fL (IQR 43.8–45.3)] and lowest in the ISR 50–69% group [43.2 fL (42.7–43.7)]. A parallel pattern was observed across Mehran class (H = 50.57, *p* < 0.001; Mehran class III–IV: 44.9 fL [44.2–45.8]). In multivariable analysis, RDW-SD independently associated with ISR ≥70% (OR = 1.228 per 0.5 fL, 95% CI 1.122–1.344, *p* < 0.001) and Mehran class III–IV (OR = 1.274, 95% CI 1.155–1.406, *p* < 0.001). Hierarchical modeling showed that adding RDW-SD improved the AUC from 0.603 to 0.719 for ISR ≥ 70% and from 0.592 to 0.757 for Mehran class III–IV (LRT *p* < 0.001 for both), with incrementally larger IDI and NRI gains for the Mehran class III–IV outcome. PDW did not retain significance after adjustment; MSI and PHR were not significantly associated with either outcome. *Conclusions*: RDW-SD was independently associated with both angiographic ISR severity and Mehran morphological complexity in patients with established drug-eluting stent restenosis, with numerically greater model discrimination for the Mehran class III–IV endpoint. These findings suggest that RDW-SD may provide complementary information regarding restenosis burden and complexity in patients with established ISR. Prospective studies are required to validate these observations and determine their clinical relevance.

## 1. Introduction

Percutaneous coronary intervention (PCI) with drug-eluting stent (DES) implantation has become a cornerstone of contemporary coronary revascularization and has substantially reduced restenosis rates compared with bare-metal stents [1,2]. Despite major advances in stent design, polymer technology, and antiplatelet therapy, in-stent restenosis (ISR) remains an important clinical challenge associated with recurrent myocardial ischemia, repeat revascularization procedures, impaired quality of life, and increased healthcare utilization [2,3].

The pathophysiology of ISR is multifactorial and involves a complex vascular reparative response triggered by endothelial injury following stent implantation. Chronic inflammation, oxidative stress, vascular smooth muscle cell proliferation, and extracellular matrix remodeling contribute to progressive luminal narrowing within the stented segment [3,4]. In later stages, neoatherosclerosis may further accelerate lesion progression and contribute to more advanced restenotic phenotypes characterized by diffuse disease, proliferative patterns, and total vessel occlusion [5,6].

Beyond the degree of luminal narrowing, angiographic morphology represents an important determinant of ISR severity. The Mehran classification provides a widely accepted framework for characterizing restenotic lesion complexity and distribution [7]. Higher Mehran classes (III–IV) have been consistently associated with more challenging revascularization procedures, higher recurrence rates, and less favorable long-term outcomes [7,8]. Accordingly, characterization of factors associated with both restenosis severity and restenotic complexity may provide clinically relevant insights into the biological mechanisms underlying advanced ISR.

Red cell distribution width (RDW), a routinely available hematological parameter reflecting erythrocyte size heterogeneity, has emerged as a potential marker of systemic inflammation and oxidative stress. Elevated RDW has been linked to adverse cardiovascular outcomes, including heart failure, acute coronary syndromes, and mortality following PCI [9,10,11]. Among the available RDW parameters, red cell distribution width–standard deviation (RDW-SD) may be less influenced by variations in mean corpuscular volume and has been proposed as a more robust measure of erythrocyte size heterogeneity in clinical research [11,12].

Platelet distribution width (PDW), a measure of platelet size variability, has emerged as an indirect marker of platelet activation and thromboinflammatory activity. Increased PDW reflects enhanced platelet turnover and heterogeneity, processes that have been linked to endothelial dysfunction, atherosclerotic progression, and adverse cardiovascular outcomes. Several studies have demonstrated associations between elevated PDW levels and greater coronary artery disease burden, impaired coronary perfusion, and unfavorable outcomes following percutaneous coronary intervention (PCI) [13,14]. Despite these observations, the relationship between PDW and the angiographic severity or morphological complexity of in-stent restenosis has not been adequately investigated.

Previous investigations evaluating hematological biomarkers in coronary intervention populations have predominantly focused on RDW and adverse clinical outcomes following PCI [15,16,17]. However, data examining the relationship between RDW-SD and the angiographic severity spectrum of ISR remain limited. Likewise, the association between RDW-SD and restenotic lesion complexity as defined by the Mehran classification has not been adequately characterized. Furthermore, comparative data regarding the relationship of RDW-SD and PDW with other exploratory laboratory-derived indices, including the Metabolic Stress Index (MSI) and the Platelet-to-High-Density Lipoprotein Ratio (PHR), are scarce in patients with established ISR [18,19].

Therefore, the present study investigated the association of RDW-SD with both angiographic restenosis severity and morphological complexity among patients undergoing repeat coronary angiography for clinically suspected DES restenosis. Restenosis severity was quantified using quantitative coronary angiography (QCA) across a broad spectrum of luminal narrowing, whereas morphological complexity was assessed according to the Mehran classification. In addition, RDW-SD was evaluated alongside PDW, MSI, and PHR to explore their respective relationships with severe restenosis and complex restenotic morphology within an established ISR population. The primary objective was to characterize associative relationships between these routinely available biomarkers and angiographic ISR burden rather than to evaluate the prediction of future restenosis occurrence.

## 2. Materials and Methods

### 2.1. Study Design and Patient Population

This was a single-center, retrospective observational study conducted at Gaziantep University Sahinbey Research and Application Hospital, a tertiary referral center with a high-volume interventional cardiology program. The study protocol was approved by the Gaziantep University Non-Interventional Clinical Research Ethics Committee (Decision No: 2026/106; 4 February 2026) and was conducted in accordance with the ethical principles of the Declaration of Helsinki. Due to the retrospective nature of the study and the use of anonymized hospital records, the requirement for individual written informed consent was waived by the Ethics Committee. The study is reported in accordance with the Strengthening the Reporting of Observational Studies in Epidemiology (STROBE) statement.

**Figure 1:**
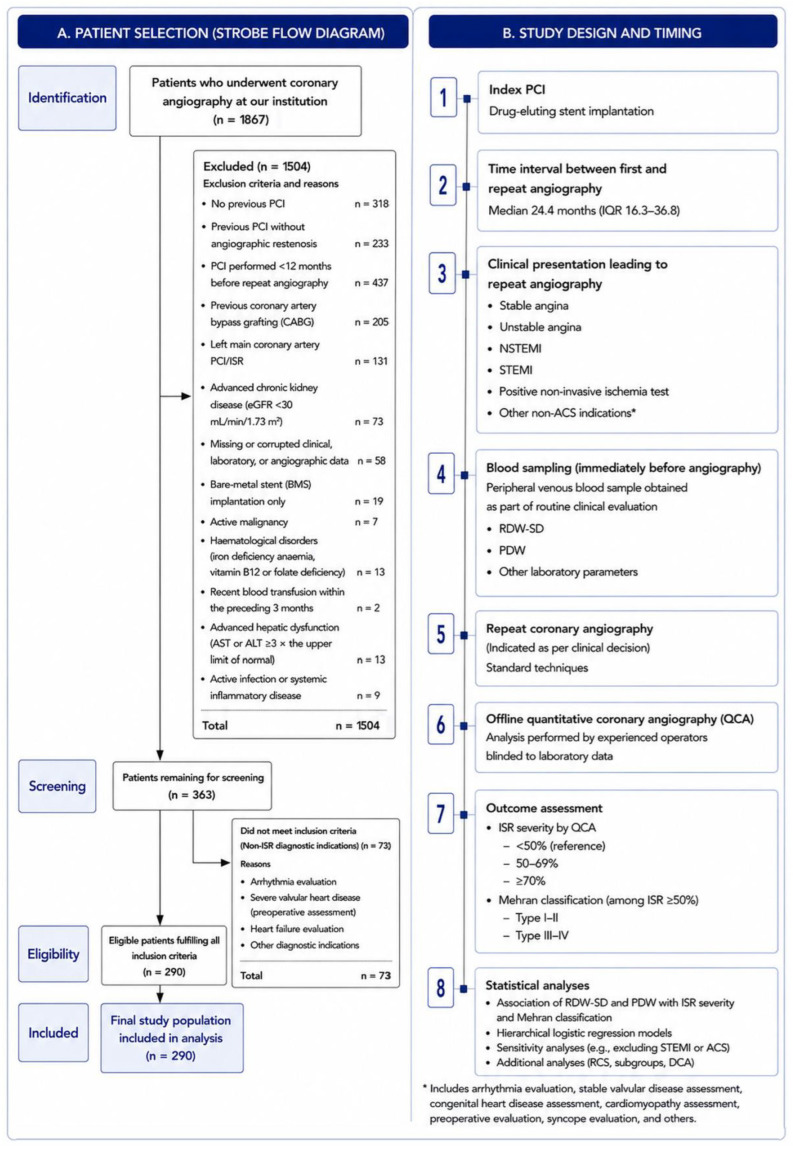
STROBE-compliant patient selection flow diagram and study design [20]. Panel (**A**) illustrates cohort derivation and application of predefined exclusion criteria. Panel (**B**) summarizes the study timeline, timing of blood sampling immediately before repeat coronary angiography, angiographic outcome assessment by QCA, and the overall analytical workflow. Abbreviations: ACS, acute coronary syndrome; BMS, bare-metal stent; CABG, coronary artery bypass grafting; CKD, chronic kidney disease; DES, drug-eluting stent; ISR, in-stent restenosis; PCI, percutaneous coronary intervention; QCA, quantitative coronary angiography; RDW-SD, red cell distribution width–standard deviation.

Electronic medical records and procedural angiographic databases were retrospectively reviewed to identify consecutive patients with a documented history of prior percutaneous coronary intervention (PCI) with drug-eluting stent (DES) implantation who subsequently underwent clinically indicated repeat coronary angiography between 1 January 2023 and 31 December 2025. The interval between index PCI and repeat coronary angiography was 24.4 months (interquartile range [IQR], 16.3–36.8 months). Patient selection and study workflow are summarized in Figure 1.

Clinical, laboratory, and procedural characteristics were retrospectively extracted from the institutional electronic medical record system and angiographic database. Collected variables included age; sex; major cardiovascular risk factors, including diabetes mellitus, arterial hypertension, and lipid profile parameters (total cholesterol, low-density lipoprotein cholesterol [LDL-C], high-density lipoprotein cholesterol [HDL-C], and triglycerides); as well as the clinical presentation prompting repeat coronary angiography. For study purposes, patients were categorized into four predefined presentation groups: (0) elevated pre-test probability of obstructive coronary artery disease or objective evidence of myocardial ischemia on non-invasive testing (including exercise stress testing, myocardial perfusion imaging, stress echocardiography, or coronary computed tomography angiography when available); (1) chronic coronary syndrome (CCS); (2) non-ST-segment elevation myocardial infarction (NSTEMI); and (3) ST-segment elevation myocardial infarction (STEMI). These baseline clinical characteristics were subsequently evaluated in relation to both angiographic restenosis severity and restenosis morphological complexity.

Patients were eligible for inclusion if they (i) were aged 18 years or older; (ii) had undergone index DES implantation at least 12 months prior to repeat angiography, to restrict the study population to established late restenotic processes driven by neointimal hyperplasia and neoatherosclerosis rather than early procedural failure; and (iii) had complete clinical, angiographic, and laboratory data available for analysis.

Patients were excluded if they had conditions predefined in the study protocol that could substantially influence hematological indices or interfere with angiographic assessment, including previous coronary artery bypass grafting, left main coronary artery intervention, bare-metal stent implantation, advanced chronic kidney disease, active malignancy, hematological disorders, recent blood transfusion, advanced hepatic dysfunction, active infection or systemic inflammatory disease, missing or corrupted clinical, laboratory, or angiographic data, or other predefined exclusion criteria. The complete patient selection process and reasons for exclusion are presented in Figure 1A.

In patients with multivessel disease or multiple restenotic segments, the clinically dominant ISR lesion defined as the lesion primarily responsible for the clinical presentation and the indication for repeat angiography was selected as the index lesion for all quantitative and morphological assessments. All analyses were conducted on a per-patient, per-index-lesion basis. After sequential application of all predefined inclusion and exclusion criteria, 290 patients with drug-eluting stent-in-stent restenosis (DES-ISR) constituted the final analytical cohort (Figure 1A).

### 2.2. Angiographic Analysis and Restenosis Classification

Coronary angiography was performed using the standard Judkins technique with a Siemens Artis Q digital angiography platform (Siemens Healthineers, Erlangen, Germany). Quantitative coronary angiography (QCA) measurements were obtained offline from digitally stored angiographic images using the integrated Siemens syngo Dynamics QCA workstation. Projections were selected to minimize vessel overlap and foreshortening. The minimal luminal diameter (MLD) and reference vessel diameter (RVD) were measured, and the percentage diameter stenosis was calculated as:

*Diameter stenosis* (%) = [(*RVD* − *MLD*)/*RVD*] × 100

All angiographic images were independently reviewed by two experienced interventional cardiologists blinded to clinical and laboratory data. In the event of disagreement, a consensus decision was reached in consultation with a third senior interventional cardiologist.

Angiographic inclusion was defined as any quantifiable luminal narrowing of 20% or greater within the stent segment or within 5 mm proximal or distal to the stent edges, assessed by QCA. The lower boundary of 20% was selected to enable biomarker evaluation across the full spectrum of restenotic change, including non-obstructive degrees of luminal loss that precede hemodynamically significant ISR. The conventional threshold of ≥50% diameter stenosis was applied to define angiographically significant ISR for the purposes of Mehran classification and clinical intervention; the 20–49% subgroup was analyzed as a reference comparator group in all between-group analyses.

Angiographic luminal narrowing was analyzed both as a continuous variable and categorized into three predefined groups: (i) reference (<50% in-stent luminal narrowing), representing subclinical restenotic change; (ii) intermediate ISR (50–69% diameter stenosis); and (iii) severe ISR (≥70% diameter stenosis). In all patients with ISR ≥ 70%, drug-eluting balloon (DEB) angioplasty was performed; balloon catheter diameter and length were therefore recorded exclusively in this subgroup.

The Mehran angiographic risk classification was calculated in all patients with ISR ≥ 50%, as this threshold defines the boundary above which the scoring algorithm is clinically applicable. Patients in the reference group (<50% in-stent luminal narrowing) were assigned a reference category for comparative purposes. For logistic regression analyses, the Mehran classification was dichotomized into low-to-intermediate risk (Mehran class I–II) and high risk (Mehran class III–IV), consistent with previously published prognostic thresholds for morphological ISR complexity. Mehran class III–IV lesions were considered complex ISR patterns owing to their diffuse and proliferative morphology.

Procedural characteristics recorded for each patient included culprit vessel (left anterior descending artery [LAD], right coronary artery [RCA], or circumflex artery [CX]), stent diameter, and stent length, obtained from procedural angiographic records. Clinical presentation categories were explored descriptively and in supplementary analyses but were not included in the primary multivariable models. Because clinical presentation is intrinsically linked to angiographic disease severity and restenosis complexity, adjustment for presentation status was considered likely to result in overadjustment and attenuation of biologically relevant associations between study biomarkers and restenosis characteristics.

### 2.3. Laboratory Parameters and Composite Index Calculations

Peripheral venous blood samples were collected immediately prior to coronary angiography as part of routine clinical evaluation, to reflect each patient’s systemic hematological and biochemical status at the time of restenosis assessment. All analyses were performed in the hospital’s central laboratory using validated automated methods.

Hematological parameters including hemoglobin, RDW-SD, platelet count, PDW, and lymphocyte count were measured using an automated hematology analyzer (Abbott CELL-DYN, Abbott Diagnostics, Chicago, IL, USA). Biochemical parameters included aspartate aminotransferase (AST), alanine aminotransferase (ALT), serum creatinine, urea, and a fasting lipid panel (total cholesterol, LDL-C, HDL-C, triglycerides).

The standard deviation variant of RDW (RDW-SD, expressed in femtolitres [fL]) was used as the primary study parameter, in preference to the coefficient of variation form (RDW-CV, expressed as a percentage). RDW-SD quantifies the absolute width of the erythrocyte volume distribution and is less susceptible to artifactual elevation in clinical contexts where mean corpuscular volume (MCV) is directionally shifted—such as iron deficiency, vitamin B12 deficiency, or folate deficiency—which may disproportionately amplify RDW-CV independently of true erythrocyte size heterogeneity. Although patients with known nutritional deficiency anemias were excluded from the study population, RDW-SD was selected a priori to minimize residual confounding from subclinical nutritional deficiencies that might remain undetected in a retrospective cardiovascular cohort.

Two investigator-derived composite indices were calculated as exploratory comparators, using exclusively routinely available laboratory parameters. MSI was designed to reflect the balance between systemic metabolic–oxidative burden and lipid-mediated cardio protection, with elevated transaminases and creatinine serving as surrogates of hepatic and renal stress, normalized against the anti-inflammatory and endothelial-protective properties of HDL-C:

*MSI* = (*ALT* + *AST* + *Creatinine*)/*HDL-C*

The Platelet-to-HDL Ratio (PHR) was calculated to index platelet-mediated thromboinflammatory activity against HDL-C-mediated endothelial protection:

*PHR* = *Platelet count*/*HDL-C*

Both indices were included for exploratory contextual comparison only; neither has undergone external validation in ISR populations, and their inclusion should be interpreted accordingly.

### 2.4. Use of Artificial Intelligence

No artificial intelligence-based tools or machine learning methods were used in the design, data analysis, interpretation, or writing of this study.

### 2.5. Statistical Analysis

All statistical analyses were performed using R statistical software (version 4.3.3; R Foundation for Statistical Computing, Vienna, Austria). The normality of continuous variables was evaluated using the Shapiro–Wilk test. As the majority of continuous variables did not satisfy the normality assumption, non-parametric methods were applied throughout. Continuous variables are presented as median and interquartile range (IQR); categorical variables are expressed as counts and percentages.

Between-group comparisons across the three angiographic severity groups (ın-stent luminal narrowing; <50%, 50–69%, and ≥70%) were performed using the Kruskal–Wallis test for continuous variables and the chi-square test for categorical variables. When overall Kruskal–Wallis tests indicated significant differences, Bonferroni-corrected pairwise post hoc comparisons were performed using the Mann–Whitney U test (adjusted significance threshold α = 0.05/3 = 0.017).

Spearman rank correlation analyses were performed to evaluate associations between study biomarkers and restenosis severity measures, including percentage diameter stenosis, severe restenosis (ISR ≥ 70%), Mehran class, and complex restenosis morphology (Mehran class III–IV). Results are reported as correlation coefficients (r) with two-tailed *p*-values.

Univariable logistic regression analyses were performed to evaluate the association of 11 candidate variables—RDW-SD (per 0.5-fL increment), PDW, hemoglobin, diabetes mellitus, hypertension, age, sex, stent length, stent diameter, MSI, and PHR—with each study outcome. Variables with *p* < 0.05 in univariable analyses together with clinically relevant covariates were entered into multivariable logistic regression models. Results are expressed as odds ratios (ORs) with 95% confidence intervals (CI). Multicollinearity was assessed using variance inflation factors (VIF); all VIF values in the full model were below 1.1, confirming absence of meaningful multicollinearity.

Hierarchical logistic regression models were constructed in four sequential blocks for each binary outcome. Model 1 included demographic confounders (age and sex). Model 2 added metabolic risk factors (diabetes mellitus and hypertension). Model 3 further added procedural parameters (stent length and stent diameter). Model 4 incorporated RDW-SD to evaluate its incremental contribution beyond all preceding covariates. Discriminative performance was quantified as the area under the ROC curve (AUC) with 95% CIs estimated by 2000-iteration bootstrap resampling. Between-model improvement was evaluated using the likelihood ratio test (LRT). RDW-SD odds ratios in hierarchical models are expressed per 0.5-fL increment.

The incremental discriminative performance after addition of RDW-SD (Model 3 versus Model 4) was formally quantified using the integrated discrimination improvement (IDI) and net reclassification improvement (NRI), both calculated with 2000-iteration bootstrap resampling. Continuous NRI reflects net probability improvement across event and non-event groups; categorical NRI was computed using the Youden-optimal predicted probability from Model 4 as the reclassification threshold (ISR ≥ 70%: 0.310; Mehran class III–IV: 0.263).

The dose–response relationship between RDW-SD and the log-odds of each outcome was visualized using restricted cubic spline (RCS) logistic regression, with three knots placed at the 10th, 50th, and 90th percentiles of the RDW-SD distribution (42.6, 43.8, and 45.8 fL, respectively). Bootstrap confidence bands were computed with 500 iterations. Clinical utility across a spectrum of decision thresholds was assessed using decision curve analysis (DCA), with net benefit plotted against threshold probability. The Youden-optimal RDW-SD cut-off was identified from ROC analysis for each outcome.

Two prespecified sensitivity analyses were performed to examine the robustness of the primary findings. In the first analysis, patients presenting with ST-segment elevation myocardial infarction (STEMI) were excluded. In the second analysis, all acute coronary syndrome (ACS) presentations (STEMI and NSTEMI) were excluded, restricting the cohort to clinically stable patients. Univariable, multivariable, and hierarchical logistic regression analyses were repeated using the same modeling strategy for both sensitivity cohorts.

A two-sided *p*-value < 0.05 was considered statistically significant for all primary analyses.

## 3. Results

### 3.1. Study Population and Baseline Characteristics

A total of 290 consecutive patients fulfilling the predefined eligibility criteria were included in the final analysis (Figure 1). All patients had angiographically confirmed drug-eluting stent-in-stent restenosis (DES-ISR). Median age was 66.0 years (IQR 58–73), and 208 patients (71.7%) were male. Angiographic luminal narrowing, quantified by quantitative coronary angiography (QCA), ranged from 20% to 100%: 111 patients (38.3%) comprised the reference group (<50% in-stent luminal narrowing), 76 (26.2%) had ISR 50–69%, and 103 (35.5%) had ISR ≥ 70%. Baseline characteristics are summarized in Table 1.

Clinical presentation differed markedly across groups (*p* < 0.001). All 38 patients with STEMI (100%) belonged to the ISR ≥ 70% group, whereas NSTEMI predominated in the ISR 50–69% group (48.7%). Stable angina (CCS) was the leading presentation in the reference group (44.1%). As expected, all patients presenting with STEMI belonged to the ISR ≥ 70% group, and most were classified as Mehran class III–IV lesions (Supplementary Table S2).

Hypertension was the only comorbidity with a significant between-group difference (30.6% vs. 44.7% vs. 51.5%; *p* = 0.007). Diabetes mellitus (approximately 46–51%), sex distribution, and age did not differ across restenosis severity groups (all *p* > 0.05). Stent diameter and length were comparable across groups (medians 3.0 mm and 24–25 mm, respectively; both *p* > 0.05).

Among patients with ISR ≥ 50%, the Mehran class distribution was markedly skewed toward higher risk in the ISR ≥ 70% group: Mehran class III was present in 27.2% and class IV in 36.9%, compared with 5.3% and 0%, respectively, in the ISR 50–69% group (*p* < 0.001). Biochemical parameters, including liver enzymes, renal function, and the full lipid panel, showed no significant between-group differences (all *p* > 0.05). The Metabolic Stress Index (MSI = [ALT + AST + Creatinine]/HDL-C) and Platelet-to-HDL Ratio (PHR = Platelet/HDL-C) were likewise similar across groups (*p* = 0.391 and 0.681, respectively).

### 3.2. RDW-SD Distribution Across Restenosis Severity and Mehran Classifications

**Figure 2:**
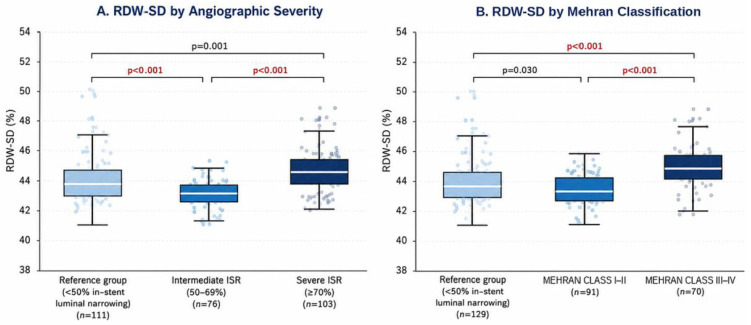
RDW-SD distribution according to angiographic luminal narrowing and Mehran classification. Panel (**A**) illustrates RDW-SD values according to angiographic luminal narrowing categories, including the reference group (<50% in-stent luminal narrowing), intermediate ISR (50–69%), and severe ISR (≥70%). Panel (**B**) presents RDW-SD values according to Mehran classification among patients with angiographically significant ISR (≥50%). Boxes represent the interquartile range, with the median indicated by the central line; whiskers denote 1.5 × IQR, and individual observations are shown as jittered points. *p* values were calculated using Bonferroni-adjusted pairwise Mann–Whitney U tests.

RDW-SD (fL) differed significantly across the three angiographic severity groups (Kruskal–Wallis H = 51.14, *p* < 0.001; Supplementary Table S3; Figure 2). The ISR 50–69% group had the lowest median RDW-SD [43.2 fL (IQR 42.7–43.7)], the reference group was intermediate [43.8 fL (43.0–44.7)], and the ISR ≥ 70% group had the highest values [44.6 fL (43.8–45.3)]. Bonferroni-corrected pairwise comparisons confirmed significant differences between all group pairs: reference group vs. ISR 50–69% (*p* < 0.001), reference group vs. ISR ≥ 70% (*p* = 0.001), and ISR 50–69% vs. ISR ≥ 70% (*p* < 0.001).

An analogous gradient was observed across Mehran classifications (H = 50.57, *p* < 0.001). Median RDW-SD values were 43.6 fL (42.9–44.6) in the reference group (<50% in-stent luminal narrowing), 43.2 fL (42.8–44.1) in Mehran class I–II, and 44.9 fL (44.2–45.8) in Mehran class III–IV. After Bonferroni correction, significant differences remained between the reference and Mehran class III–IV groups and between the Mehran class I–II and Mehran class III–IV groups, whereas the difference between the reference and Mehran class I–II groups was no longer statistically significant (adjusted *p* = 0.090).

### 3.3. Spearman Rank Correlations

RDW-SD showed the strongest and most consistent associations with ISR-related variables among all study biomarkers (Supplementary Table S1). Point-biserial correlations with the binary outcomes were r = 0.347 (*p* < 0.001) for ISR ≥ 70% and r = 0.389 (*p* < 0.001) for Mehran class III–IV. PDW showed a weak negative correlation with Mehran class III–IV only (r = −0.129, *p* = 0.028) and was not significantly associated with ISR ≥ 70% (*p* = 0.180). MSI and PHR showed no significant correlation with either outcome (all *p* > 0.05).

### 3.4. Univariable and Multivariable Logistic Regression

#### 3.4.1. Outcome: ISR ≥ 70%

Eleven candidate variables were entered into univariable logistic regression (Table 2). RDW-SD, expressed per 0.5-fL increment, was the strongest univariable associate of ISR ≥ 70% (OR = 1.224, 95% CI 1.120–1.337, *p* < 0.001). Hypertension was the only other variable to reach significance (OR = 1.855, 95% CI 1.139–3.022, *p* = 0.013). PDW, MSI, hemoglobin, DM, age, sex, stent length, stent diameter, and PHR did not reach statistical significance in univariable analysis. The two significant variables were entered into the multivariable model, in which both retained independent significance: RDW-SD per 0.5-fL increment (OR = 1.228, 95% CI 1.122–1.344, *p* < 0.001) and hypertension (OR = 1.919, 95% CI 1.151–3.198, *p* = 0.012; McFadden R^2^ = 0.079; AUC = 0.718, 95% CI 0.620–0.816).

#### 3.4.2. Outcome: Mehran Class III–IV

For the Mehran class III–IV outcome, univariable analysis identified three variables showing significant univariable associations (Table 3): RDW-SD per 0.5-fL increment (OR = 1.279, 95% CI 1.162–1.408, *p* < 0.001), PDW (OR = 0.847, 95% CI 0.735–0.975, *p* = 0.021), and hypertension (OR = 1.815, 95% CI 1.055–3.123, *p* = 0.031). In the multivariable model, RDW-SD retained strong independent significance (OR = 1.274, 95% CI 1.155–1.406, *p* < 0.001) and hypertension remained significant (OR = 1.873, 95% CI 1.047–3.353, *p* = 0.035). PDW was attenuated after mutual adjustment and did not reach significance (OR = 0.867, *p* = 0.062; McFadden R^2^ = 0.120; AUC = 0.763, 95% CI 0.670–0.856).

### 3.5. Hierarchical Logistic Regression and Discriminative Performance

Sequential hierarchical models were constructed by stepwise addition of covariate blocks to a demographic base (Table 4 and Table 5). Across both outcomes, Models 1–3 (covering demographic, metabolic, and procedural covariates) produced negligible-to-modest discrimination. For ISR ≥ 70%, the AUC ranged from 0.524 in Model 1 to 0.603 in Model 3; for Mehran class III–IV, the AUC ranged from 0.546 to 0.592. Neither the addition of DM and hypertension (Model 2) nor the addition of stent dimensions (Model 3) reached likelihood ratio test (LRT) significance for the Mehran class III–IV outcome (*p* = 0.108 and 0.813, respectively).

**Figure 3:**
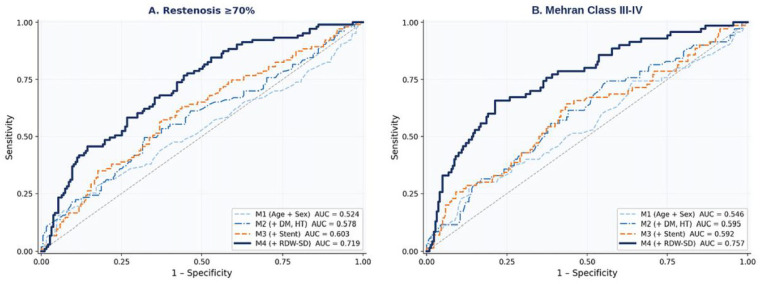
Receiver operating characteristic curves for sequential hierarchical models. Panel (**A**): ISR ≥ 70% (*n* = 103 events). Panel (**B**): Mehran class III–IV (*n* = 70 events). M1: age + sex. M2: M1 + DM + HT. M3: M2 + stent dimensions. M4: M3 + RDW-SD. Diagonal dashed grey line: reference (AUC = 0.500). AUC values are shown in the legend for each model. Abbreviations: AUC, area under the receiver operating characteristic curve; DM, diabetes mellitus; HT, hypertension; ISR, in-stent restenosis; RDW-SD, red cell distribution width–standard deviation.

The addition of RDW-SD in Model 4 produced a significant improvement in model performance. For ISR ≥ 70%: LRT χ^2^(1) = 24.83, *p* < 0.001; AUC increased from 0.603 to 0.719 (95% CI 0.610–0.827); McFadden R^2^ rose from 0.022 to 0.088; and per-0.5-fL OR was 1.238 (*p* < 0.001). For Mehran class III–IV, LRT χ^2^(1) = 31.41, *p* < 0.001; AUC increased from 0.592 to 0.757 (95% CI 0.641–0.872); McFadden R^2^ rose from 0.019 to 0.117; and per-0.5-fL OR was 1.294 (*p* < 0.001; Figure 3). Variance inflation factors for all Model 4 variables were below 1.1 across both outcomes, confirming the absence of multicollinearity. These findings indicate incremental discrimination rather than predictive capability.

### 3.6. Incremental Discrimination: IDI and NRI

As an exploratory analysis, integrated discrimination improvement (IDI) and net reclassification improvement (NRI) were calculated to evaluate the incremental discrimination provided by RDW-SD beyond the base hierarchical model (Supplementary Table S4). For ISR ≥ 70%, the IDI was 0.085 (95% CI 0.055–0.117, *p* < 0.001), continuous NRI was 0.853 (95% CI 0.629–1.067, *p* < 0.001), and categorical NRI was 0.179 (95% CI 0.055–0.313, *p* = 0.006). For the Mehran class III–IV outcome, all three metrics were larger: IDI = 0.115 (95% CI 0.076–0.152, *p* < 0.001), continuous NRI = 0.931 (95% CI 0.680–1.160, *p* < 0.001), and categorical NRI = 0.318 (95% CI 0.172–0.474, *p* < 0.001). Categorical NRI thresholds were set at the Youden-optimal predicted probability of Model 4: 0.310 for ISR ≥ 70% and 0.263 for Mehran class III–IV.

### 3.7. Optimal Threshold and Non-Linear Dose–Response

Exploratory receiver operating characteristic (ROC) analyses identified a Youden-optimal RDW-SD threshold of 44.2 fL for both study outcomes. At this threshold, sensitivity and specificity were 69.9% and 74.3% for ISR ≥ 70%, and 75.7% and 69.5% for Mehran class III–IV, respectively.

**Figure 4:**
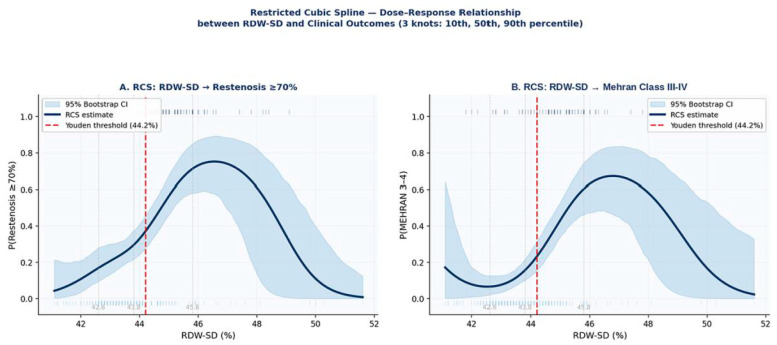
Restricted cubic spline—dose–response relationship between RDW-SD and clinical outcomes. Panel (**A**): ISR ≥ 70%. Panel (**B**): Mehran class III–IV. Three knots at the 10th, 50th, and 90th percentiles of the RDW-SD distribution (42.6, 43.8, 45.8 fL). Solid curve: spline estimate; shaded band: 95% bootstrap CI (500 iterations). Dashed red vertical line: Youden-optimal threshold (44.2 fL). Dotted grey verticals: knot positions. Rug marks: individual values (upper margin, events; lower margin, non-events). Abbreviations: CI, confidence interval; fL, femtolitres; ISR, in-stent restenosis; RDW-SD, red cell distribution width–standard deviation.

The dose–response relationship between RDW-SD and the probability of each outcome was modeled by restricted cubic spline (RCS) logistic regression with three knots positioned at the 10th, 50th, and 90th percentiles of the RDW-SD distribution (42.6, 43.8, and 45.8 fL, respectively; Figure 4). For both outcomes, the spline demonstrated a monotonically increasing non-linear probability curve rising sharply above approximately 44 fL, consistent with the empirical Youden threshold. Bootstrap 95% confidence intervals (500 iterations) widened at both tails of the distribution, reflecting sparse data at extreme RDW-SD values. These threshold estimates should be considered exploratory and hypothesis-generating, and require external validation before clinical application.

### 3.8. Clinical Utility: Decision Curve Analysis

Exploratory decision curve analysis suggested that both the RDW-SD univariable model and the multivariable model provided net clinical benefit superior to the treat-all and treat-none reference strategies across a broad range of clinically plausible threshold probabilities (Supplementary Figure S1). For ISR ≥ 70%, net benefit exceeded zero across threshold probabilities of approximately 0.15–0.45, with the multivariable model providing marginally greater benefit than RDW-SD alone at intermediate thresholds (0.25–0.40). For the Mehran class III–IV outcome, net benefit remained positive across threshold probabilities of approximately 0.10–0.35, with curves for both models tracking closely throughout this range.

### 3.9. Sensitivity Analyses

To evaluate the robustness of the primary findings, two prespecified sensitivity analyses were performed by excluding patients presenting with ST-segment elevation myocardial infarction (STEMI) and, subsequently, all acute coronary syndrome (ACS) presentations (Supplementary Tables S5A,B and S6A,B).

After exclusion of patients with STEMI, the association between RDW-SD and severe ISR (≥70%) was attenuated following multivariable adjustment (adjusted OR per 0.5-fL increase: 1.230, 95% CI 1.01–1.49, *p* = 0.037), although sequential incorporation of RDW-SD continued to improve model discrimination (AUC 0.645, 95% CI 0.566–0.719; likelihood ratio test [LRT] χ^2^ = 4.27, *p* = 0.039). In contrast, the association with complex ISR according to the Mehran classification remained robust (adjusted OR 2.637, 95% CI 1.65–4.22, *p* < 0.001), with a marked improvement in model discrimination (AUC 0.742, 95% CI 0.639–0.847; LRT χ^2^ = 19.74, *p* < 0.001) (Supplementary Table S5A,B).

Similarly, exclusion of all ACS presentations yielded findings consistent with the primary analysis. RDW-SD remained independently associated with severe ISR (adjusted OR 1.470, 95% CI 1.14–1.89, *p* = 0.003) and complex ISR (adjusted OR 5.166, 95% CI 2.29–11.67, *p* < 0.001). Addition of RDW-SD to the sequential models resulted in improved discrimination for severe ISR (AUC 0.731, 95% CI 0.638–0.810; LRT χ^2^ = 9.22, *p* = 0.002) and complex ISR (AUC 0.820, 95% CI 0.715–0.908; LRT χ^2^ = 23.35, *p* < 0.001) (Supplementary Table S6A,B).

## 4. Discussion

In this retrospective observational study of patients with DES-related in-stent restenosis, higher RDW-SD values were independently associated with increasing angiographic restenosis severity and lesion complexity according to the Mehran classification. Sequential hierarchical modeling demonstrated that incorporation of RDW-SD provided incremental improvement in model discrimination beyond conventional clinical and procedural variables. These associations remained broadly consistent across prespecified sensitivity analyses, supporting the robustness of the observed associations. In contrast, PDW, MSI, and PHR did not retain independent associations after multivariable adjustment.

RDW reflects the coefficient of heterogeneity of erythrocyte volume distribution and is sensitive to disruptions in erythropoiesis arising from a range of systemic stressors. Several biological mechanisms may contribute to the observed associations. Chronic low-grade systemic inflammation, a central driver of neointimal hyperplasia and neoatherosclerosis following coronary stent implantation, is known to impair erythrocyte maturation through cytokine-mediated suppression of erythropoietin signaling and iron reutilization, resulting in increased size heterogeneity of circulating red cells [9,10]. Oxidative stress, which is also implicated in restenotic vascular remodeling, disrupts the erythrocyte membrane and accelerates red cell turnover, independently contributing to RDW elevation [9]. Endothelial dysfunction, a shared upstream pathway in both ISR pathobiology and elevated RDW, may further link the two phenomena through impaired vascular homeostasis and amplified inflammatory signaling [3,4]. Neurohormonal activation and autonomic dysregulation accompanying acute coronary syndromes may also contribute to RDW elevation through bone marrow adrenergic stimulation and accelerated erythrocyte release [9,11]. Collectively, these pathways suggest that RDW-SD functions not as a disease-specific ISR marker, but rather as an integrative hematological surrogate of systemic biological stress that appears to be associated with the severity of vascular reparative failure at the site of prior coronary stenting [10,11,16,17].

Incremental discrimination analyses consistently suggested a stronger association between RDW-SD and restenosis complexity than with angiographic stenosis severity alone. Although these differences were modest, they support the concept that complex ISR morphology may better reflect the cumulative biological processes underlying vascular reparative failure. This observation is supported by the consistently greater improvement in model discrimination for the Mehran class III-IV endpoint than for ISR ≥ 70%, including higher AUC (0.757 vs. 0.719), IDI (0.115 vs. 0.085), continuous NRI (0.931 vs. 0.853), and categorical NRI (0.318 vs. 0.179) following incorporation of RDW-SD into the hierarchical models. The Mehran class III–IV pattern may reflect more advanced neointimal proliferation, diffuse vascular remodeling, and neoatherosclerotic change than focal ISR lesions, potentially indicating a more sustained inflammatory and reparative response [5,6,7,12]. This biological gradient may be more faithfully captured by an integrative hematological marker such as RDW-SD than by a single angiographic diameter measurement, which does not distinguish between focal hemodynamically significant ISR and diffuse proliferative occlusive disease. Accordingly, RDW-SD appears to be more closely associated with morphologically complex restenotic disease than with luminal stenosis severity alone, although this hypothesis requires prospective external validation [7,8,13]. The prespecified sensitivity analyses further strengthened the interpretation of the primary results. After exclusion of STEMI, the association between RDW-SD and severe ISR was attenuated but remained statistically significant, albeit borderline, after multivariable adjustment, whereas the association with Mehran class III-IV lesions remained more robustly statistically significant. Moreover, exclusion of all acute coronary syndrome presentations yielded findings that were broadly consistent with the primary analyses (Supplementary Tables S5A and S6B), indicating that the observed associations were not solely driven by acute clinical presentation. The attenuation of the association between RDW-SD and ISR ≥ 70% after STEMI exclusion should be interpreted cautiously. STEMI is associated with a marked acute-phase systemic inflammatory response that independently elevates RDW-SD through cytokine-mediated erythropoietic suppression and accelerated erythrocyte release; its exclusion therefore simultaneously removes a biologically plausible source of confounding and reduces the number of events available for the ISR ≥ 70% outcome (from 103 to 65 events), diminishing statistical power. The near-unity lower bound of the confidence interval after STEMI exclusion (OR 1.230, 95% CI 1.01–1.49) is consistent with this power loss rather than with a spurious primary association. Importantly, the association with Mehran class III-IV morphological complexity remained robust after both STEMI and full ACS exclusion, suggesting that the relationship between RDW-SD and complex restenotic disease is less dependent on the acute inflammatory milieu accompanying ACS presentations. These results directly address the potential confounding effect of ACS and support the robustness of the association between RDW-SD and restenotic lesion complexity.

The ROC analyses, exploratory cut-off value, incremental discrimination metrics (IDI/NRI), and decision curve analyses should be interpreted as measures of model discrimination within the present cohort rather than evidence of predictive performance. External validation in independent prospective populations will be required before any clinical application can be considered.

The comparator indices evaluated in this study did not demonstrate consistent independent associations with either primary outcome. PDW showed a borderline association with Mehran class III-IV lesions in univariable analysis (OR = 0.847, *p* = 0.021), but this association was attenuated after multivariable adjustment (*p* = 0.062). Prior studies have reported associations between PDW and coronary flow impairment, with elevated PDW reflecting enhanced platelet turnover and thromboinflammatory activity [13,14]; however, the present findings suggest that PDW does not independently contribute to restenosis severity or morphological complexity after adjustment for RDW-SD, which may reflect a stronger signal from the erythrocytic compartment in the specific biological context of established ISR. MSI and PHR were not associated with either outcome in univariable analyses and therefore were not retained in the final multivariable models. Collectively, these findings suggest that RDW-SD may capture biological processes more closely related to restenotic lesion burden and complexity than platelet-based or exploratory composite laboratory indices [9,12,18,19].

The median interval of 24.4 months between index PCI and repeat angiography suggests that the present cohort predominantly represents late DES-ISR, a stage in which neointimal maturation and neoatherosclerosis are believed to contribute substantially to restenosis development. However, because RDW-SD was measured at the time of repeat angiography, the present findings should be interpreted as cross-sectional associations and do not establish temporal or causal relationships.

Taken together, the primary and sensitivity analyses consistently suggest that RDW-SD is more closely associated with restenotic lesion complexity, particularly higher Mehran classification, than with luminal stenosis severity alone. These findings support the concept that RDW-SD may reflect the biological processes underlying complex vascular repair rather than simply the angiographic extent of luminal narrowing.

### Limitations

Several limitations of the present study should be acknowledged when interpreting the findings. First, the retrospective single-center design precludes causal inference and introduces the possibility of residual confounding by unmeasured variables. Selection bias cannot be excluded, as only patients with sufficient clinical concern to warrant repeat coronary angiography were included, which may enrich the population towards more symptomatic or angiographically advanced ISR phenotypes. In addition, lesions with 20–49% diameter stenosis were included to permit evaluation across the full spectrum of angiographic restenotic change. Accordingly, patients with <50% diameter stenosis were analyzed as a reference group with subclinical in-stent luminal narrowing rather than clinically significant ISR, consistent with contemporary angiographic definitions. Although this approach allowed assessment of biomarker relationships across increasing restenosis severity, this subgroup does not fulfill conventional definitions of clinically significant ISR and may limit direct comparison with studies restricted to angiographically significant ISR. Second, pharmacological data, including use of statins, dual antiplatelet therapy, angiotensin-converting enzyme inhibitors, and other cardioprotective agents, were not systematically incorporated as covariates in the regression models. Statin therapy and antiplatelet agents may independently influence RDW-SD through their anti-inflammatory effects and, in the case of statins, erythropoiesis-modifying properties; the absence of medication adjustment represents a potential source of residual confounding [9,10]. Given the high background prevalence of statin and dual antiplatelet use in a post-PCI cohort, this limitation is unlikely to be differential across ISR severity groups, as statin prescription rates would be expected to be similar across all restenosis categories but systematic medication data would be required to confirm this assumption. Future prospective studies should incorporate detailed pharmacological covariate adjustment to clarify the independent contribution of RDW-SD beyond medication effects. Third, only a single RDW-SD measurement was available for each patient, obtained at the time of the index repeat angiography. Dynamic changes in RDW-SD over the post-PCI period which may be more informative regarding the biological trajectory of neointimal proliferation could not be evaluated. Fourth, although the median interval between index DES implantation and repeat angiography was 24.4 months (IQR 16.3–36.8), suggesting that the cohort predominantly represents late DES-ISR driven by neointimal maturation and neoatherosclerosis, individual time-to-repeat-angiography varied substantially across patients. The wide interquartile range reflects the heterogeneity of clinical indications and referral patterns in a retrospective real-world cohort, and precluded inclusion of time-from-PCI as a covariate in the primary regression models; doing so would have introduced a variable with biologically uncertain directionality relative to RDW-SD and ISR severity. Early and late ISR may be driven by distinct biological mechanisms; early restenosis predominantly reflecting neointimal hyperplasia and late restenosis incorporating neoatherosclerotic change and differential RDW-SD behavior across these phases cannot be excluded. Because RDW-SD was measured only at the time of repeat angiography, temporal changes in biomarker levels throughout the restenosis progression trajectory could not be evaluated. Future studies should incorporate time-from-PCI as a formal covariate and stratify analyses by early versus late ISR to address this limitation. Fifth, intravascular imaging optical coherence tomography or intravascular ultrasound was not performed, precluding histological characterization of the restenotic tissue and mechanistic correlation between RDW-SD and specific restenotic tissue patterns such as fibrin deposition, lipid infiltration, or neoatherosclerosis [6]. Sixth, smoking status was not available in the database for inclusion in covariate adjustment, despite tobacco exposure being an established determinant of both erythrocyte morphology and ISR risk [4]. Finally, although RDW-SD improved model discrimination, the observed AUC values should not be interpreted as evidence supporting clinical implementation or standalone diagnostic performance. The ROC analyses, exploratory cut-off value, IDI, NRI, and decision curve analyses should be regarded as hypothesis-generating and require prospective external validation in independent cohorts. These findings should therefore be interpreted as hypothesis-generating and exploratory within the context of an established ISR population, rather than as evidence for clinical implementation. External validation in independent, prospectively designed cohorts with complete covariate adjustment and longer follow-up would be required before any translational implications could be considered [11,15].

## 5. Conclusions

Higher RDW-SD values were independently associated with both increasing angiographic luminal narrowing and morphological complexity in patients with established DES-ISR. Among the hematological indices evaluated, RDW-SD demonstrated the most consistent association across the primary, hierarchical, and prespecified sensitivity analyses. Although these findings do not support the use of RDW-SD as a standalone diagnostic or predictive biomarker, they suggest that this readily available hematological parameter may provide complementary information regarding restenotic lesion complexity and angiographic severity. Prospective multicenter studies incorporating serial biomarker assessment and external validation are warranted to confirm these observations.

## Figures and Tables

**Table 1:** Baseline characteristics by restenosis severity group.

Variable	Reference Group (<50% In-Stent Luminal Narrowing) *n* = 111	ISR 50–69% *n* = 76	ISR ≥ 70% *n* = 103	*p*-Value
Demographic & Clinical Characteristics				
Age, years	65.0 (59.0–72.0)	66.0 (58.8–72.2)	67.0 (58.0–76.0)	0.692
Male sex, *n* (%)	75 (67.6%)	59 (77.6%)	74 (71.8%)	0.324
Diabetes mellitus, *n* (%)	51 (45.9%)	37 (48.7%)	52 (50.5%)	0.799
Hypertension, *n* (%)	34 (30.6%)	34 (44.7%)	53 (51.5%)	**0.007**
Clinical Presentation				**<0.001**
PTP/Non-invasive ischemia	43 (38.7%)	11 (14.5%)	21 (20.4%)	
CCS (stable angina)	49 (44.1%)	28 (36.8%)	19 (18.4%)	
NSTEMI	19 (17.1%)	37 (48.7%)	25 (24.3%)	
STEMI	0 (0.0%)	0 (0.0%)	38 (36.9%)	
Culprit Vessel				0.116
LAD	64 (57.7%)	34 (44.7%)	46 (44.7%)	
RCA	33 (29.7%)	26 (34.2%)	38 (36.9%)	
CX	14 (12.6%)	16 (21.1%)	19 (18.4%)	
Mehran Classification				**<0.001**
Class I	-	32 (42.1%)	8 (7.8%)	
Class II	-	22 (28.9%)	29 (28.2%)	
Class III	-	4 (5.3%)	28 (27.2%)	
Class IV	-	0 (0.0%)	38 (36.9%)	
Procedural Parameters				
Stent diameter, mm	3.0 (2.9–3.5)	3.0 (3.0–3.5)	3.0 (2.8–3.5)	0.393
Stent length, mm	25.0 (18.0–33.0)	24.0 (18.0–32.2)	24.0 (18.0–32.0)	0.829
Hematological Parameters				
Hemoglobin, g/dL	14.3 (12.9–15.3)	14.1 (13.0–15.2)	13.9 (12.9–15.1)	0.705
RDW-SD, (fL)	43.8 (43.0–44.7)	43.2 (42.7–43.7)	44.6 (43.8–45.3)	**<0.001**
PDW, %	12.3 (10.9–13.9)	12.0 (11.2–13.7)	12.0 (11.1–13.1)	0.405
Platelet, ×10^3^/μL	250.0 (208.5–296.5)	255.0 (222.0–299.0)	244.0 (201.0–293.0)	0.369
Lymphocyte, ×10^3^/μL	2.2 (1.7–2.7)	2.2 (1.7–2.6)	2.2 (1.6–2.9)	0.847
Biochemistry				
Creatinine, mg/dL	0.9 (0.7–1.0)	0.9 (0.8–1.0)	0.9 (0.7–1.0)	0.918
Urea, mg/dL	34.0 (28.0–45.0)	35.0 (28.0–44.0)	34.0 (29.0–42.5)	0.976
AST, U/L	23.0 (20.0–26.0)	23.0 (20.0–26.0)	23.0 (19.5–26.5)	0.899
ALT, U/L	20.0 (16.5–23.0)	20.0 (15.0–24.2)	21.0 (16.5–26.0)	0.385
Lipid Profile				
Total cholesterol, mg/dL	196.2 (164.9–240.2)	191.1 (154.4–236.9)	186.4 (158.0–216.7)	0.192
Triglycerides, mg/dL	151.0 (107.0–234.0)	152.0 (114.0–225.0)	141.0 (103.0–208.0)	0.499
LDL-C, mg/dL	113.0 (96.0–148.0)	107.5 (88.0–150.2)	115.0 (90.5–134.5)	0.272
HDL-C, mg/dL	45.0 (39.0–48.5)	44.0 (41.0–49.2)	43.0 (37.0–47.5)	0.326
Composite Indices				
MSI	1.0 (0.8–1.2)	1.0 (0.8–1.2)	1.0 (0.8–1.3)	0.391
PHR	5.5 (4.5–6.8)	5.9 (4.9–7.0)	5.9 (4.4–7.2)	0.681

Data presented as median (IQR) for continuous variables and *n* (%) for categorical variables. Between-group comparisons: Kruskal–Wallis test (continuous) and chi-square test (categorical). Bold *p*-values indicate statistical significance (*p* < 0.05). MEHRAN Classification was calculable only for patients with ≥50% stenosis; those with <50% not calculated). Between-group comparisons across the three angiographic severity groups were performed using the Kruskal–Wallis test for continuous variables and the chi-square test for categorical variables. Reference group; <50% in-stent luminal narrowing. Bold *p*-values denote statistical significance at *p* < 0.05. Abbreviations: ALT, alanine aminotransferase; AST, aspartate aminotransferase; CCS, chronic coronary syndrome; CX, left circumflex artery; DM, diabetes mellitus; HDL-C, high-density lipoprotein cholesterol; HT, arterial hypertension; ISR, in-stent restenosis; LAD, left anterior descending artery; LDL-C, low-density lipoprotein cholesterol; MSI, Metabolic Stress Index; NSTEMI, non-ST-segment elevation myocardial infarction; PDW, platelet distribution width; PHR, Platelet-to-HDL Ratio; PTP, pre-test probability; QCA, quantitative coronary angiography; RCA, right coronary artery; RDW-SD, red cell distribution width–standard deviation; STEMI, ST-segment elevation myocardial infarction.

**Table 2:** Univariable and multivariable logistic regression—outcome: restenosis ≥ 70%.

Variable	Univariable		Multivariable	
	OR (95% CI)	*p*	OR (95% CI)	*p*
RDW-SD (per 0.5-unit increase)	1.224 (1.120–1.337)	<0.001	1.228 (1.122–1.344)	<0.001
PDW (per unit)	0.897 (0.796–1.011)	0.075	—	—
Hemoglobin (per g/dL)	0.974 (0.849–1.116)	0.700	—	—
Diabetes mellitus	1.147 (0.709–1.856)	0.576	—	—
Hypertension	1.855 (1.139–3.022)	0.013	1.919 (1.151–3.198)	0.012
Age (per year)	1.008 (0.984–1.031)	0.527	—	—
Male sex	1.009 (0.591–1.722)	0.973	—	—
Stent length (per mm)	0.994 (0.966–1.023)	0.704	—	—
Stent diameter (per mm)	0.712 (0.394–1.287)	0.261	—	—
MSI (per unit)	1.521 (0.961–2.407)	0.074	—	—
PHR (per unit)	0.989 (0.885–1.104)	0.840	—	—
Multivariable: RDW-SD + Hypertension | McFadden R^2^ = 0.079 | AUC = 0.718 (95%CI 0.620–0.816)				

Eleven candidate variables evaluated in univariable analysis; those reaching *p* < 0.05 were entered into the multivariable model (—: not entered). RDW-SD expressed per 0.5-fL increment. All VIF values < 1.1. Abbreviations: CI, confidence interval; fL, femtolitres; HT, hypertension; ISR, in-stent restenosis; MSI, Metabolic Stress Index; OR, odds ratio; PDW, platelet distribution width; PHR, Platelet-to-HDL Ratio; RDW-SD, red cell distribution width–standard deviation; VIF, variance inflation factor.

**Table 3:** Univariable and multivariable logistic regression, outcome: MEHRAN Class III–IV.

Variable	Univariable		Multivariable	
	OR (95% CI)	*p*	OR (95% CI)	*p*
RDW-SD (per 0.5-unit increase)	1.279 (1.162–1.408)	<0.001	1.274 (1.155–1.406)	<0.001
PDW (per unit)	0.847 (0.735–0.975)	0.021	0.867 (0.746–1.007)	0.062
Hemoglobin (per g/dL)	0.941 (0.809–1.095)	0.432	—	—
Diabetes mellitus	1.274 (0.743–2.183)	0.379	—	—
Hypertension	1.815 (1.055–3.123)	0.031	1.873 (1.047–3.353)	0.035
Age (per year)	1.014 (0.988–1.041)	0.283	—	—
Male sex	0.981 (0.541–1.780)	0.950	—	—
Stent length (per mm)	1.000 (0.969–1.033)	0.979	—	—
Stent diameter (per mm)	0.821 (0.424–1.588)	0.557	—	—
MSI (per unit)	1.479 (0.948–2.308)	0.084	—	—
PHR (per unit)	1.002 (0.886–1.133)	0.973	—	—
Multivariable: RDW-SD + PDW + Hypertension | McFadden R^2^ = 0.120 | AUC = 0.763 (95%CI 0.670–0.856)				

Variable entry as in Table 2. PDW achieved univariable significance but was attenuated after adjustment (*p* = 0.062) and did not retain independent significance. RDW-SD expressed per 0.5-fL increment. All VIF values < 1.1. Abbreviations: CI, confidence interval; fL, femtolitres; HT, hypertension; OR, odds ratio; PDW, platelet distribution width; RDW-SD, red cell distribution width–standard deviation; VIF, variance inflation factor.

**Table 4:** Incremental performance of hierarchical logistic regression models for severe in-stent restenosis (ISR ≥ 70%).

Model	Variables Included	Adjusted OR (95% CI) *	*p*-Value	Pseudo-R^2^	AIC	AUC (95% CI)	ΔLRT (χ^2^, *p*)
Model 1	Age + Sex	—	—	0.001	382.9	0.524 (0.404–0.644)	—
Model 2	Model 1 + DM + HT	HT: 1.838 (1.13–2.99)	0.017	0.017	380.9	0.578 (0.459–0.696)	χ^2^ = 6.01, *p* = 0.050
Model 3	Model 2 + Stent length + Stent diameter	—	—	0.022	383.0	0.603 (0.485–0.720)	χ^2^ = 1.89, *p* = 0.389
Model 4	Model 3 + RDW-SD	1.238 (1.122–1.344)	<0.001	0.088	360.2	0.719 (0.610–0.827)	χ^2^ = 24.83, *p* < 0.001

Model 1 included age and sex. Model 2 additionally incorporated diabetes mellitus (DM) and hypertension (HT). Model 3 further included procedural variables (stent length and stent diameter). Model 4 incorporated RDW-SD (per 0.5-fL increment) to evaluate its incremental contribution to model discrimination for severe in-stent restenosis (ISR ≥ 70%). Model performance was assessed using the Akaike Information Criterion (AIC), McFadden’s pseudo-R^2^, and the area under the receiver operating characteristic curve (AUC). Differences between sequential models were evaluated using likelihood ratio tests (LRTs), with each model compared against the immediately preceding model. The 95% confidence intervals for AUCs were estimated using 2000 bootstrap resamples. Multicollinearity was assessed using variance inflation factors (VIF); all VIF values were <1.10 in the final model, indicating negligible collinearity. Abbreviations: AIC, Akaike Information Criterion; AUC, area under the receiver operating characteristic curve; CI, confidence interval; DM, diabetes mellitus; HT, hypertension; ISR, in-stent restenosis; LRT, likelihood ratio test; OR, odds ratio; RDW-SD, red cell distribution width–standard deviation; VIF, variance inflation factor. * Adjusted odds ratios (ORs) are presented only for variables retained in each hierarchical model. RDW-SD is expressed per 0.5-fL increment.

**Table 5:** Incremental performance of hierarchical logistic regression models for complex in-stent restenosis according to the Mehran classification.

Model	Variables Included	Adjusted OR (95% CI) *	*p*-Value	Pseudo-R^2^	AIC	AUC (95% CI)	ΔLRT (χ^2^, *p*)
Model 1	Age + Sex	—	—	0.004	325.4	0.546 (0.412–0.680)	—
Model 2	Age + Sex + DM + HT	HT: 1.738 (0.99–3.04)	0.051	0.018	324.9	0.595 (0.463–0.727)	χ^2^ = 4.44, *p* = 0.108
Model 3	Model 2 + Stent length + Stent diameter	—	—	0.019	328.5	0.592 (0.460–0.724)	χ^2^ = 0.42, *p* = 0.813
Model 4	Model 3 + RDW-SD	1.294 (1.155–1.406)	<0.001	0.117	299.1	0.757 (0.641–0.872)	χ^2^ = 31.41, *p* < 0.001

The hierarchical modeling strategy was identical to that used in Table 4. Model 1 included age and sex. Model 2 additionally incorporated diabetes mellitus (DM) and hypertension (HT). Model 3 further included procedural variables (stent length and stent diameter). Model 4 incorporated RDW-SD (per 0.5-fL increment) to evaluate its incremental contribution to model discrimination for complex in-stent restenosis (Mehran class III–IV). Model performance was assessed using the Akaike Information Criterion (AIC), McFadden’s pseudo-R^2^, and the area under the receiver operating characteristic curve (AUC). Differences between sequential models were evaluated using likelihood ratio tests (LRTs), with each model compared against the immediately preceding model. The 95% confidence intervals for AUCs were estimated using 2000 bootstrap resamples. Multicollinearity was assessed using variance inflation factors (VIF); all VIF values were <1.10 in the final model, indicating negligible collinearity. Abbreviations: AIC, Akaike Information Criterion; AUC, area under the receiver operating characteristic curve; CI, confidence interval; DM, diabetes mellitus; HT, hypertension; LRT, likelihood ratio test; OR, odds ratio; RDW-SD, red cell distribution width–standard deviation; VIF, variance inflation factor. * Adjusted odds ratios (ORs) are presented only for variables retained in each hierarchical model. RDW-SD is expressed per 0.5-fL increment.

## Data Availability

The datasets generated and/or analyzed during the current study are not publicly available due to institutional data protection policies but are available from the corresponding author on reasonable request.

## Supplementary Materials

The following supporting information can be downloaded at: https://www.mdpi.com/article/10.3390/medicina62071358/s1. Table S1: Spearman Correlations Between Continuous Study Variables and Clinical Outcomes; Table S2: Association of Clinical Presentation With Severe Restenosis and Mehran Class III–IV; Table S3: RDW-SD Distribution by Restenosis Severity and Mehran Classification; Table S4: Incremental Discrimination Metrics: M3 → M4 (Adding RDW-SD); Table S5: Sensitivity Analysis 1: Excluding STEMI, including Table S5A: Incremental Performance of Sequential Logistic Regression Models for Severe In-Stent Restenosis (ISR ≥ 70%) and Table S5B: Incremental Performance of Sequential Logistic Regression Models for Complex In-Stent Restenosis According to the Mehran Classification; Table S6: Sensitivity Analysis 2: Excluding ACS, including Table S6A: Incremental Performance of Sequential Logistic Regression Models for Severe In-Stent Restenosis (ISR ≥ 70%) and Table S6B: Incremental Performance of Sequential Logistic Regression Models for Complex In-Stent Restenosis According to the Mehran Classification; Figure S1: Decision Curve Analysis.

## Abbreviations

AUC	Area Under the Receiver Operating Characteristic Curve
CCS	Canadian Cardiovascular Society (stable angina)
CI	Confidence Interval
DCA	Decision Curve Analysis
DES	Drug-Eluting Stent
DM	Diabetes Mellitus
HT	Arterial Hypertension
IDI	Integrated Discrimination Improvement
IQR	Interquartile Range
ISR	In-Stent Restenosis
IVUS	Intravascular Ultrasound
LRT	Likelihood Ratio Test
MEHRAN	Mehran Angiographic Restenosis Classification
MSI	Metabolic Stress Index
NRI	Net Reclassification Improvement
NSTEMI	Non-ST-Segment Elevation Myocardial Infarction
OCT	Optical Coherence Tomography
OR	Odds Ratio
PCI	Percutaneous Coronary Intervention
PDW	Platelet Distribution Width
PHR	Platelet-to-High-Density Lipoprotein Ratio
PTP	Pre-Test Probability
QCA	Quantitative Coronary Angiography
RCS	Restricted Cubic Spline
RDW-SD	Red Cell Distribution Width–Standard Deviation
STEMI	ST-Segment Elevation Myocardial Infarction
VIF	Variance Inflation Factor
