# Supplementary material for: Red Cell Distribution Width–Standard Deviation and the Severity of In-Stent Restenosis: Associations with Angiographic Stenosis Burden and Mehran Classification
Source: Medicina (Kaunas). 2026 Jul 14;62(7):1358. doi: 10.3390/medicina62071358 (PMC13414383; doi:10.3390/medicina62071358)
Supplement: Supplementary file 1 [file medicina-62-01358-s001.zip › Supplementary Figure.pdf]

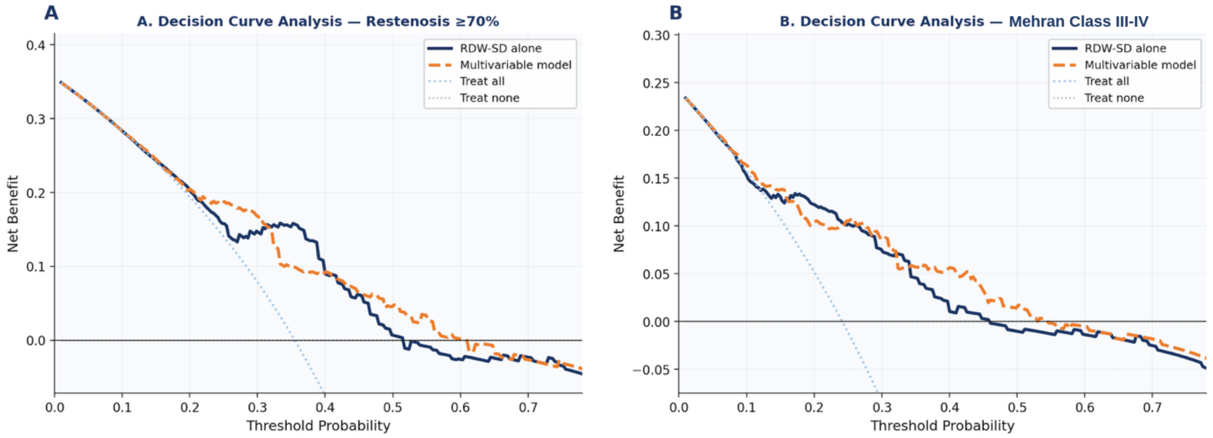

Supplementary Figure S1. Decision Curve Analysis : Panel A: ISR ≥70%. Panel B: Mehran Class III-IV. Solid dark blue: RDW-SD model. Dashed orange: multivariable model (RDW-SD + HT for ISR ≥70%; RDW-SD + PDW + HT for Mehran Class III-IV). Dotted light blue: treat-all. Dotted grey (net benefit = 0): treat-none.

Abbreviations: HT, hypertension; ISR, in-stent restenosis; PDW, platelet distribution width; RDW-SD, red cell distribution width–standard deviation.
