# Supplementary material for: Red Cell Distribution Width–Standard Deviation and the Severity of In-Stent Restenosis: Associations with Angiographic Stenosis Burden and Mehran Classification
Source: Medicina (Kaunas). 2026 Jul 14;62(7):1358. doi: 10.3390/medicina62071358 (PMC13414383; doi:10.3390/medicina62071358)
Supplement: Supplementary file 1 [file medicina-62-01358-s001.zip › SupplementaryTables.pdf]

Supplementary Table S1 Spearman Correlations Between Continuous Study Variables and Clinical Outcomes

Variable	Restenosis ≥70% r (p)	MEHRAN CLASS III-IV r (p)
**RDW-SD**	**0.347 (<0.001)**	**0.389 (<0.001)**
PDW	-0.079 (0.180)	-0.129 (0.028)
Hemoglobin	-0.046 (0.436)	-0.083 (0.161)
MSI	0.080 (0.172)	0.045 (0.446)
PHR	0.007 (0.901)	0.031 (0.596)
Age	0.048 (0.413)	0.074 (0.207)
Stent length	-0.021 (0.722)	-0.008 (0.898)
Stent diameter	-0.064 (0.280)	-0.033 (0.572)

Point-biserial Spearman coefficients (r) with two-tailed p-values. No correction for multiple comparisons applied; exploratory analysis.

Abbreviations: *ISR, in-stent restenosis; MSI, Metabolic Stress Index; PDW, platelet distribution width; PHR, Platelet-to-HDL Ratio; RDW-SD, red cell distribution width–standard deviation.*

Supplementary Table S2. Association of Clinical Presentation With Severe Restenosis and Mehran Class III-IV

Clinical Presentation	Total *n*	Restenosis ≥70% *n* (%)	Mehran Class III-IV *n* (%)
PTP / Non-invasive ischemia	75	21 (28.0)	12 (16.0)
CCS	96	19 (19.8)	13 (13.5)
NSTEMI	81	25 (30.9)	10 (12.3)
STEMI	38	38 (100.0)	35 (92.1)
**Overall test**	—	χ^2 = 81.97	χ^2 = 110.61
**Effect size**	—	Cramer's V = 0.532	Cramer's V = 0.618
**p-value**	—	**<0.001**	**<0.001**

Pearson chi-square test. Effect size: Cramér's V. Restenosis ≥70% and Mehran Class III-IV were analyzed as binary outcomes.
Abbreviations: *CCS, chronic coronary syndrome; ISR, in-stent restenosis; NSTEMI, non-ST-segment elevation myocardial infarction; PTP, pre-test probability; STEMI, ST-segment elevation myocardial infarction.*

Supplementary Table S3. RDW-SD Distribution by Restenosis Severity and Mehran Classification

Group	Median RDW-SD (IQR)	Overall p (Kruskal–Wallis)	Post-hoc comparisons (Bonferroni-corrected
**Restenosis Severity Group**	**(H = 51.14, overall p < 0.001)**		
<50% (Reference)	43.8 (43.0–44.7)	**<0.001**	<50% vs 50–69%: p < 0.001
50–69%	43.2 (42.7–43.7)	—	<50% vs ≥70%: p = 0.001

≥70%	44.6 (43.8–45.3)	—	50–69% vs ≥70%: p < 0.001
**Mehran Classification (H = 50.57, overall p < 0.001)**			
Reference (<50% stenosis)	43.6 (42.9–44.6)	<0.001	Reference vs Mehran Class I-II: p = 0.030
Mehran Class I-II	43.2 (42.8–44.1)	—	Reference vs Mehran Class III-IV: p < 0.001
Mehran Class III-IV	44.9 (44.2–45.8)	—	Mehran Class I-II vs Class III-IV: p < 0.001

Pairwise: Mann–Whitney U with Bonferroni correction (α/3 = 0.017). Kruskal–Wallis H-statistics in group headers. ***Abbreviations:*** *fL, femtolitres; IQR, interquartile range; ISR, in-stent restenosis; RDW-SD, red cell distribution width–standard deviation.*

Methodological Note: MSI in Regression Analysis

In univariable analysis, MSI showed a borderline non-significant association with restenosis ≥70% (OR=1.52, p=0.074). In the prior multivariable model (which included all 11 predictors simultaneously), MSI appeared significant (p=0.034) due to a suppression effect: Hypertension acted as a negative confounder, and mutual adjustment altered the partial regression coefficients such that MSI's association was magnified. This is a known statistical phenomenon (negative confounding/suppressor variable) rather than a genuine independent clinical signal. In the current revised analysis, MSI does not enter the multivariable model because it did not reach p<0.05 in univariable testing. Authors are advised not to interpret MSI as an independent predictor without replication in a dedicated study.

Supplementary Table S4 Incremental Discrimination Metrics: M3 → M4 (Adding RDW-SD)

Metric	M3 AUC<br>Base model (Age+Sex+DM+HT +Stent)	M4 AUC<br>M3 + RDW-SD	Estimate (95% Bootstrap CI)<br>M3 → M4 incremental improvement	p
**Outcome 1: Restenosis ≥70% (n cases = 103, n controls = 187)**				
IDI	0.603	0.719	0.0854 (0.0545 – 0.1171)	**<0.001**
NRI (continuous)			0.853 (0.629 – 1.067)	**<0.001**
NRI (categorical)			0.179 (0.055 – 0.313)	**0.006**
**Outcome 2:** Mehran Class III-IV **(n cases = 70, n controls = 220)**				
IDI	0.592	0.757	0.1146 (0.0758 – 0.1520)	**<0.001**
NRI (continuous)			0.931 (0.680 – 1.160)	**<0.001**
NRI (categorical)			0.318 (0.172 – 0.474)	**<0.001**

AUC values shown for M3 and M4 models. M3: Age + Sex + DM + HT + Stent length + Stent diameter. M4: M3 + RDW-SD.

2,000-iteration bootstrap resampling. Categorical NRI threshold: Youden-optimal predicted probability of M4.

Abbreviations: AUC, area under the ROC curve; CI, confidence interval; IDI, integrated discrimination improvement; ISR, in-stent restenosis; NRI, net reclassification improvement; RDW-SD, red cell distribution width–standard deviation.

Supplementary Table S5A. Incremental Performance of Sequential Logistic Regression Models for Severe In-Stent Restenosis (ISR ≥70%)— Sensitivity Analysis 1: Excluding STEMI (Non-STEMI Cohort; n = 252)

Model	Variables Included	Adjusted OR (95% CI)*	p-value	Pseudo-R^2	AIC	AUC (95% CI)	ΔLRT (χ^2, p)
**Model 1**	Age + Sex	—	—	0.002	293.0	0.523 (0.436–0.607)	—
**Model 2**	Model 1 + DM + HT	HT: 1.884 (1.05–3.36)	**0.032**	0.019	292.4	0.600 (0.520–0.681)	χ^2=4.64, p=0.098
**Model 3**	Model 2 + Stent length + Stent diameter	—	—	0.027	294.1	0.612 (0.536–0.690)	χ^2=2.31, p=0.315

**Model 4**	Model 3 + RDW-SD	**RDW-SD:1.230 (1.01–1.49)**	**0.037**	0.041	291.8	**0.645 (0.566–0.719)**	**χ^2=4.27, p=0.039**

Model 1 included age and sex. Model 2 additionally incorporated diabetes mellitus (DM) and hypertension (HT). Model 3 further included procedural variables (stent length and stent diameter). Model 4 incorporated RDW-SD (per 0.5-fL increment) to evaluate its incremental contribution to model discrimination. Model performance was assessed using McFadden's pseudo-R^2, the Akaike Information Criterion (AIC), and the area under the ROC curve (AUC). Differences between sequential models were evaluated using likelihood ratio tests (LRT). AUC 95% confidence intervals estimated using 2,000 bootstrap resamples. Multicollinearity was assessed using variance inflation factors (VIF); all VIF values were <1.10 in the final model.

Abbreviations: AIC, Akaike Information Criterion; AUC, area under the receiver operating characteristic curve; CI, confidence interval; DM, diabetes mellitus; HT, hypertension; ISR, in-stent restenosis; LRT, likelihood ratio test; OR, odds ratio; RDW-SD, red cell distribution width–standard deviation; VIF, variance inflation factor. Only variables remaining independently associated with the outcome are presented in the "Adjusted OR (95% CI)" column. Complete multivariable regression coefficients are available in the primary analysis (Table 2).

Supplementary Table S5B. Incremental Performance of Sequential Logistic Regression Models for Complex In-Stent Restenosis According to the Mehran Classification— Sensitivity Analysis 1: Excluding STEMI (ISR ≥50% Subgroup; n = 141)

Model	Variables Included	Adjusted OR (95% CI)*	p-value	Pseudo-R^2	AIC	AUC (95% CI)	ΔLRT (χ^2, p)
**Model 1**	Age + Sex	—	—	0.000	164.0	0.502 (0.393–0.621)	—
**Model 2**	Model 1 + DM + HT	—	—	0.005	167.3	0.533 (0.432–0.640)	χ^2=0.70, p=0.706
**Model 3**	Model 2 + Stent length + Stent diameter	—	—	0.006	171.1	0.565 (0.457–0.673)	χ^2=0.25, p=0.883
**Model 4**	Model 3 + RDW-SD	**RDW-SD : 2.637 (1.65–4.22)**	**<0.001**	0.131	153.3	**0.742 (0.639–0.847)**	**χ^2=19.74, p<0.001**

Model 1 included age and sex. Model 2 additionally incorporated diabetes mellitus (DM) and hypertension (HT). Model 3 further included procedural variables (stent length and stent diameter). Model 4 incorporated RDW-SD (per 0.5-fL increment) tto evaluate its incremental contribution to model discrimination. Model performance was assessed using McFadden's pseudo-R^2, the Akaike Information Criterion (AIC), and the area under the ROC curve (AUC). Differences between sequential models were evaluated using likelihood ratio tests (LRT). AUC 95% confidence intervals estimated using 2,000 bootstrap resamples. Multicollinearity was assessed using variance inflation factors (VIF); all VIF values were <1.10 in the final model.

Abbreviations: AIC, Akaike Information Criterion; AUC, area under the receiver operating characteristic curve; CI, confidence interval; DM, diabetes mellitus; HT, hypertension; ISR, in-stent restenosis; LRT, likelihood ratio test; OR, odds ratio; RDW-SD, red cell distribution width–standard deviation; VIF, variance inflation factor. Only variables remaining independently associated with the outcome are presented in the "Adjusted OR (95% CI)" column. Complete multivariable regression coefficients are available in the primary analysis (Table 2).

Supplementary Table S6A. Incremental Performance of Sequential Logistic Regression Models for Severe In-Stent Restenosis (ISR ≥70%)— Sensitivity Analysis 2: Excluding ACS (Stable CAD + CCS Cohort; n = 171)

Model	Variables Included	Adjusted OR (95% CI)*	p- value	Pseudo-R^2	AIC	AUC (95% CI)	ΔLRT (χ^2, p)
**Model 1**	Age + Sex	—	—	0.019	188.5	0.579 (0.477–0.672)	—
**Model 2**	Model 1 + DM + HT	—	—	0.034	189.7	0.635 (0.537–0.721)	χ^2=2.80, p=0.247
**Model 3**	Model 2 + Stent length + Stent diameter	—	—	0.057	189.5	0.671 (0.570–0.760)	χ^2=4.19, p=0.123
**Model 4**	Model 3 + RDW-SD	**RDW-SD:1.470 (1.14–1.89)**	**0.003**	0.106	182.3	**0.731 (0.638–0.810)**	**χ^2=9.22, p=0.002**

Model 1 included age and sex. Model 2 additionally incorporated diabetes mellitus (DM) and hypertension (HT). Model 3 further included procedural variables (stent length and stent diameter). Model 4 incorporated RDW-SD (per 0.5-fL increment) to evaluate its incremental contribution to model discrimination. Model performance was assessed using McFadden's pseudo-R^2, the Akaike Information Criterion (AIC), and the area under the ROC curve (AUC). Differences between sequential models were evaluated using likelihood ratio tests (LRT). AUC 95% confidence intervals estimated using 2,000 bootstrap resamples. Multicollinearity was assessed using variance inflation factors (VIF); all VIF values were <1.10 in the final model.

Abbreviations: AIC, Akaike Information Criterion; AUC, area under the receiver operating characteristic curve; CI, confidence interval; DM, diabetes mellitus; HT, hypertension; ISR, in-stent restenosis; LRT, likelihood ratio test; OR, odds ratio; RDW-SD, red cell distribution width–standard deviation; VIF, variance inflation factor. Only variables remaining independently associated with the outcome are presented in the "Adjusted OR (95% CI)" column. Complete multivariable regression coefficients are available in the primary analysis (Table 2).

Supplementary Table S6B. Incremental Performance of Sequential Logistic Regression Models for Complex In-Stent Restenosis According to the Mehran Classification—

Sensitivity Analysis 2: Excluding ACS (ISR ≥50% Subgroup; n = 79)

Model	Variables Included	Adjusted OR (95% CI)*	p-value	Pseudo-R^2	AIC	AUC (95% CI)	ΔLRT (χ^2, p)
**Model 1**	Age + Sex	—	—	0.001	104.5	0.495 (0.343–0.640)	—
**Model 2**	Model 1 + DM + HT	—	—	0.006	108.0	0.523 (0.375–0.667)	χ^2=0.48, p=0.785
**Model 3**	Model 2 + Stent length + Stent diameter	—	—	0.012	111.5	0.527 (0.383–0.658)	χ^2=0.56, p=0.755
**Model 4**	Model 3 + RDW-SD	**RDW-SD: 5.166 (2.29–11.67)**	**<0.001**	0.248	90.1	**0.820 (0.715–0.908)**	**χ^2=23.35, p<0.001**

Model 1 included age and sex. Model 2 additionally incorporated diabetes mellitus (DM) and hypertension (HT). Model 3 further included procedural variables (stent length and stent diameter). Model 4 incorporated RDW-SD (per 0.5-fL increment) to evaluate its incremental contribution to model discrimination. Model performance was assessed using McFadden's pseudo-R^2, the Akaike Information Criterion (AIC), and the area under the ROC curve (AUC). Differences between sequential models were evaluated using likelihood ratio tests (LRT). AUC 95% confidence intervals estimated using 2,000 bootstrap resamples. Multicollinearity was assessed using variance inflation factors (VIF); all VIF values were <1.10 in the final model.

Abbreviations: AIC, Akaike Information Criterion; AUC, area under the receiver operating characteristic curve; CI, confidence interval; DM, diabetes mellitus; HT, hypertension; ISR, in-stent restenosis; LRT, likelihood ratio test; OR, odds ratio; RDW-SD, red cell distribution width–standard deviation; VIF, variance inflation factor. Only variables remaining independently associated with the outcome are presented in the "Adjusted OR (95% CI)" column. Complete multivariable regression coefficients are available in the primary analysis (Table 2).
